# CanDrA: Cancer-Specific Driver Missense Mutation Annotation with Optimized Features

**DOI:** 10.1371/journal.pone.0077945

**Published:** 2013-10-30

**Authors:** Yong Mao, Han Chen, Han Liang, Funda Meric-Bernstam, Gordon B. Mills, Ken Chen

**Affiliations:** 1 Department of Bioinformatics and Computational Biology, The University of Texas M.D. Anderson Cancer Center, Houston, Texas, United States of America; 2 Department of Surgical Oncology, The University of Texas M.D. Anderson Cancer Center, Houston, Texas, United States of America; 3 Department of Systems Biology, The University of Texas M.D. Anderson Cancer Center, Houston, Texas, United States of America; Karolinska Institutet, Sweden

## Abstract

Driver mutations are somatic mutations that provide growth advantage to tumor cells, while passenger mutations are those not functionally related to oncogenesis. Distinguishing drivers from passengers is challenging because drivers occur much less frequently than passengers, they tend to have low prevalence, their functions are multifactorial and not intuitively obvious. Missense mutations are excellent candidates as drivers, as they occur more frequently and are potentially easier to identify than other types of mutations. Although several methods have been developed for predicting the functional impact of missense mutations, only a few have been specifically designed for identifying driver mutations. As more mutations are being discovered, more accurate predictive models can be developed using machine learning approaches that systematically characterize the commonality and peculiarity of missense mutations under the background of specific cancer types. Here, we present a cancer driver annotation (CanDrA) tool that predicts missense driver mutations based on a set of 95 structural and evolutionary features computed by over 10 functional prediction algorithms such as CHASM, SIFT, and MutationAssessor. Through feature optimization and supervised training, CanDrA outperforms existing tools in analyzing the glioblastoma multiforme and ovarian carcinoma data sets in The Cancer Genome Atlas and the Cancer Cell Line Encyclopedia project.

## Introduction

Cancer is a complex genetic disease. The occurrence and progression of most cancers can be attributed to accumulated mutations in the cancer genome [1]. At different stages of oncogenesis, a group of key mutations, called drivers, significantly alter the normal cellular system [2], [3] and confer growth and survival advantages to tumor cells [4]. However, due to the inherent genomic instability present in tumors, driver mutations occur on the background of a large number of mutations, called passengers, that are not functionally related to oncogenesis. The identification of driver mutations is a critical mission of cancer genomics. A few drivers have been identified and are widely used as diagnostic and/or prognostic biomarkers, or as drug targets for cancer treatment [5], [6]. Research that interrogate specific driver mutations and their clinical implications are being widely conducted for multiple types of cancer [7], [8], however, more efforts are demanded for systematic genome-wide characterization of driver mutations and their functional implications.

The majority of mutations detected in cancer are point mutations. When occurring in coding regions of genes, they may alter protein-coding sequences, affect protein structure and expression, or disrupt protein-protein interactions [9]. Mutations that alter amino acid sequences are called non-synonymous mutations, among which the majority are missense mutations that substitute amino acid residues. Unlike frame-shift or nonsense mutations, which usually lead to truncated proteins, the function of missense mutations is less obvious. Nonetheless, a large number of missense mutations have been demonstrated as drivers, such as the *BRAF* V600E mutation in melanoma [10], and *KRAS* G12D and G12V mutations in colorectal cancer [11].

The rarity and low prevalence of driver mutations make them extremely difficult to predict using conventional statistical methods that require moderate sample sizes [1], [12]–[14]. Much of the data sparseness can be attributed to a high degree of genetic heterogeneity underlying clinically defined cancer types. Moreover, the function of a missense mutation may be dependent on many other factors that are variable under different conditions, such as genetic predisposition, presence of other somatic mutations, cell lineage, and stage of malignancy.

In recent years, multiple computational methods have been proposed for evaluating the functional impact of missense mutations. Collectively, these methods have computed more than 90 relevant quantities or features that describe the properties of a mutation and its associated site from the aspects of (a) evolutionary conservation, (b) physicochemical properties of the proteins, (c) protein domains, and (d) sequence context. Different methods may utilize these four types of features individually or in combination. In particular, MutationAssessor [9] and SIFT [15] use type (a) features, SNPs3D uses types (a) and (b), CanPredict [16] uses types (a) and (c), MutationTaster [17] and SNAP [18] use types (a), (b), and (c), and CHASM [19] and PolyPhen 2 [20] use all four types of features.

Most of these methods were designed to solve a general genetic problem, i.e., discriminating deleterious mutations from non-deleterious ones. However, most of the algorithms do not consider the specific genetic or disease context in which a mutation occurs. Although they can be applied to assess somatic missense mutations, the results clearly lack specificity [13], [14], [19]. Since driver mutations are defined under a specific disease context, a driver mutation prediction method would not be accurate without taking into consideration disease-specific factors such as cancer type, disease stage, mutation prevalence, mutation spectrum, and other clinical characteristics.

Among the published methods, CHASM is the only one that explicitly considers cancer-type-specific factors [19]. In CHASM, 86 different features from all four feature types are used to characterize each missense mutation, and the classification models are trained in a cancer-type-specific fashion using a random forest algorithm. The training data for a cancer type include a set of curated driver mutations as positive examples and a nearly equal number of synthetic passenger mutations (SPMs) as negative examples.

Although CHASM represents a considerable advance in predicting driver mutations, a few caveats exist. First, it is not clear whether the SPMs are sufficient of modelling the broad spectrum of passenger mutations that occur. Further, recent evidence has indicated that the occurrence of passenger mutations is affected by definable factors, e.g., sequence context, replication timing, and gene expression, that are likely not sufficiently represented by the set of random SPMs [21], [22]. Second, recent methods have generated new predictive features [9], [23]–[26] that were not considered in the development of the CHASM algorithm. Third, it is unclear whether the random forest algorithm is optimal given the relatively small size of the training set and the high-dimensionality of the data sets to be analyzed. Fourth, the large amount of mutation data accumulated from recent large-scale cancer genome sequencing projects and community based projects including clinical sequencing have not been sufficiently integrated into CHASM to improve the predictive power.

Due to these considerations, we aimed to assess whether more accurate driver mutation predictions can be achieved by systematically integrating the large amount of newly available data and existing algorithms. We started by performing a comprehensive analysis of mutation data in the COSMIC database [27], The Cancer Genome Atlas (TCGA), and the Cancer Cell Line Encyclopedia (CCLE) project [28] and derived sets of training and test data for supervised model training and evaluation. We performed a thorough analysis of the existing tools to compare and select the most effective features. Our efforts resulted in a new cancer driver annotation tool, CanDrA, that integrates our curated data and features to compute a driver score for each possible missense mutation in a specific human cancer type. We demonstrated that CanDrA achieved better sensitivity and specificity than other tools in predicting driver mutations in glioblastoma multiforme (GBM) and ovarian carcinoma (OVC). CanDrA and the associated datasets for major cancer types (e.g., breast, colorectal, malignant melanoma, and squamous cell skin cancer) are available at http://bioinformatics.mdanderson.org/main/CanDrA.

## Materials and Methods

### Data Curation

#### The stringent set (S)

Two missense mutation datasets, GBM and OVC, were curated from those reported in COSMIC (V58), TCGA, and the CCLE project. TCGA data contained a total of 727 mutations from 142 GBM samples and 11,005 mutations from 316 OVC samples [13], [14]. The COSMIC data contained 640 mutations from 351 GBM primary tumor samples and 237 from 212 OVC primary tumor samples. We defined a driver mutation as one that was observed in at least two different samples, from either TCGA or COSMIC. To be stringent, we excluded recurrent mutations that coincided with other putative functional mutations such as indels, nonsense mutations, nonstop mutations, splice site mutations, and translation start site mutations in the same gene of the same sample. Those overlapping with dbSNP sites were also excluded. This process resulted in 67 driver mutations for GBM and 61 for OVC, most (92.5% and 80.3%, respectively) of which had been regarded as drivers in previous studies [19].

We selected passenger mutations from hyper-mutated samples, which have deficiency in DNA damage repairing and have much higher fractions of passenger mutations than non-hyper-mutated samples [14]. Three GBM samples were identified from TCGA, each with over 55 missense mutations, and two OVC samples were identified, each with over 130 mutations. A candidate was excluded if it was located in any cancer gene (as defined by the COSMIC cancer census or by the CHASM study), or overlapped with dbSNP. Finally, 95 and 246 mutations were respectively selected for GBM and OVC. We also curated a second set of passenger mutations from the CCLE project, which contains mutations from 27 GBM cell lines and 19 OVC cell lines. After applying the same criteria, 490 mutations for GBM and 462 mutations for OVC were selected.

In summary, four stringent sets were formed: GBM.S1, GBM.S2, OVC.S1 and OVC.S2 (Table 1 and Tables S1–S4 in File S1). These sets were used as independent test sets to measure CanDrA’s performance against those of other tools.

**Table 1:** Datasets used for training and testing CanDrA.

Datasets	Cancer Type	#Drivers	#Passengers	Used for
GBM.Ex	GBM	1529 (COSMIC)	1259 (COSMIC)	Training
GBM.S1	GBM	67 (TCGA+COSMIC)	95 (TCGA)	Test
GBM.S2	GBM	67 (TCGA+COSMIC)	490 (CCLE)	Test
OVC.Ex	OVC	1768 (COSMIC)	8075 (COSMIC)	Training
OVC.S1	OVC	61 (TCGA+COSMIC)	246 (TCGA)	Test
OVC.S2	OVC	61 (TCGA+COSMIC)	462 (CCLE)	Test

Listed are the numbers of driver and passenger mutations for glioblastoma multiforme (GBM) and ovarian carcinoma (OVC) curated from the Catalogue of Somatic Mutations in Cancer (COSMIC), The Cancer Genome Atlas (TCGA), and the Cancer Cell Line Encyclopedia (CCLE).

#### The expanded set (E)

Many mutations occur recurrently in close proximity (hotspots) in different types of cancer. For example, the *BRAF* V600 mutation occurs in papillary thyroid carcinoma, colorectal cancer, melanoma and non-small-cell lung cancer, as do *BRAF* N580S, E585K, D593V, F594L, G595R, L596V, T598I, V599D, V599E, V599K, V599R, K600E, and A727V mutations. Most of these mutations are clustered in two hotspot regions: the glycine-rich P loop of the N lobe and the activation segment and flanking regions [29]. Many similar hotspot mutations are observed in *TP53, PIK3CA, KRAS,* amongst others [30], [31]. These mutations have similar properties and likely have similar functions in different cancer types. To represent such commonality across cancer types, we constructed a cancer-type-specific but expanded set of drivers and passengers using the following empirical rules.

For a given cancer type, we call a missense mutation a driver mutation if it occurs in a gene mutated in this cancer type and 1) it is observed in at least 3 primary tumor samples (regardless of cancer type), or 2) its site intersects at least 4 mutations (including indels, dinucleotide or trinucleotide mutations), or 3) it is centered in a 25 bp region that intersects at least 5 mutations in the COSMIC database. We subtracted driver mutations in set S from this set to ensure their mutual independence. This process resulted in 1529 and 1768 putative drivers for GBM and OVC, respectively.

Passenger mutations of a cancer type were chosen as those that occur only once in primary tumor samples of this cancer type, not in any COSMIC cancer census gene, and do not coincide with any other mutations within a 31-bp window in the entire COSMIC database. We also subtracted passenger mutations in set S from this set. This process resulted in 1259 and 8075 passengers for GBM and OVC, respectively (Table 1).

By combining these putative drivers and passengers for each cancer type, two expanded datasets were formed: GBM.Ex and OVC.Ex. They were used as our training sets for feature selection and supervised training.

### Descriptive Features

For each missense mutation, 95 features (Table S5 in File S1) were acquired from four data portals: CHASM’s SNVBOX [19], ENSEMBL Variant Effect Predictor [32], Mutation Assessor [9] and ANNOVAR [33]. Among them are UniProtKB annotations, evolutionary conservation scores, protein physicochemical properties, sequence context indices, and functional impact scores computed by algorithms such as SIFT [15], PolyPhen-2 [20], CONDEL [25], Mutation Assessor [9], PhyloP [26], GERP++ [24] and LRT [23].

### Feature Selection and Evaluation

A small fraction around 6.0% of data was not available from these data portals. SNVBOX missed about 13.3% data in 29 features because there is no related Uniprot protein domain information for some mutation sites. ANNOVAR missed around 15% data in features such as Phylop, Gerp++ and LRT scores due to unknown reasons. To facilitate our investigation, we substituted the missing features with those of the nearest mutations in the same gene using a k-nearest neighbor algorithm. Our evaluation was minimally affected by this operation because our selected test sets were almost free of missing features.

We evaluated the predictive performance of each feature based on the Mann–Whitney U test and the area under the curve (AUC) of the receiver operating characteristic curve. Features with non-significant *p* values after Bonferroni correction and AUCs below a specified threshold were excluded from further analysis; as were a few features that may introduce dataset (population)-specific biases (e.g., AACOSMIC). We then assessed feature combinations using a hybrid feature selection algorithm. First, all possible combinations with fewer than 4 selected features were enumerated and evaluated based on the average AUCs from 10-fold cross-validation (repeated 5 times) on the training dataset. Second, the best feature combination was further expanded using a hill-climbing search strategy [34], which iteratively included the remaining features into the current combination. The feature set that achieved the maximum AUC in cross-validation was selected as the optimal set.

### Classification Results and Scores

We use a weighted support vector machine (SVM) [35] as our classifier in order to address the imbalanced numbers of drivers and passengers in the training set. CanDrA classifies a mutation into 3 categories: driver, no-call, and passenger, based on scores computed by the SVM (Figure S1 in File S1) [36]. According to the score distributions, a mutation is classified as a driver if its score is greater than the 90^th^ percentile of those of the passenger mutations in the training set, as a passenger if its score is less than the 10^th^ percentile of those of the driver mutations, or as a no-call otherwise. In addition, CanDrA computes a confidence score for each prediction, defined as the fraction of mutations that have more extreme scores in the same class in the training data (Figure S1 in File S1). For example, if a mutation is classified as a driver and its score is greater than those of 95% of the drivers in the training set, its confidence score is equal to 0.05. These confidence scores are thus *de facto* significance *P* values estimated from the empirical class-wise score distribution in the training dataset.

## Results

### Feature Selection and Overall Classification Results

**Figure 1:**
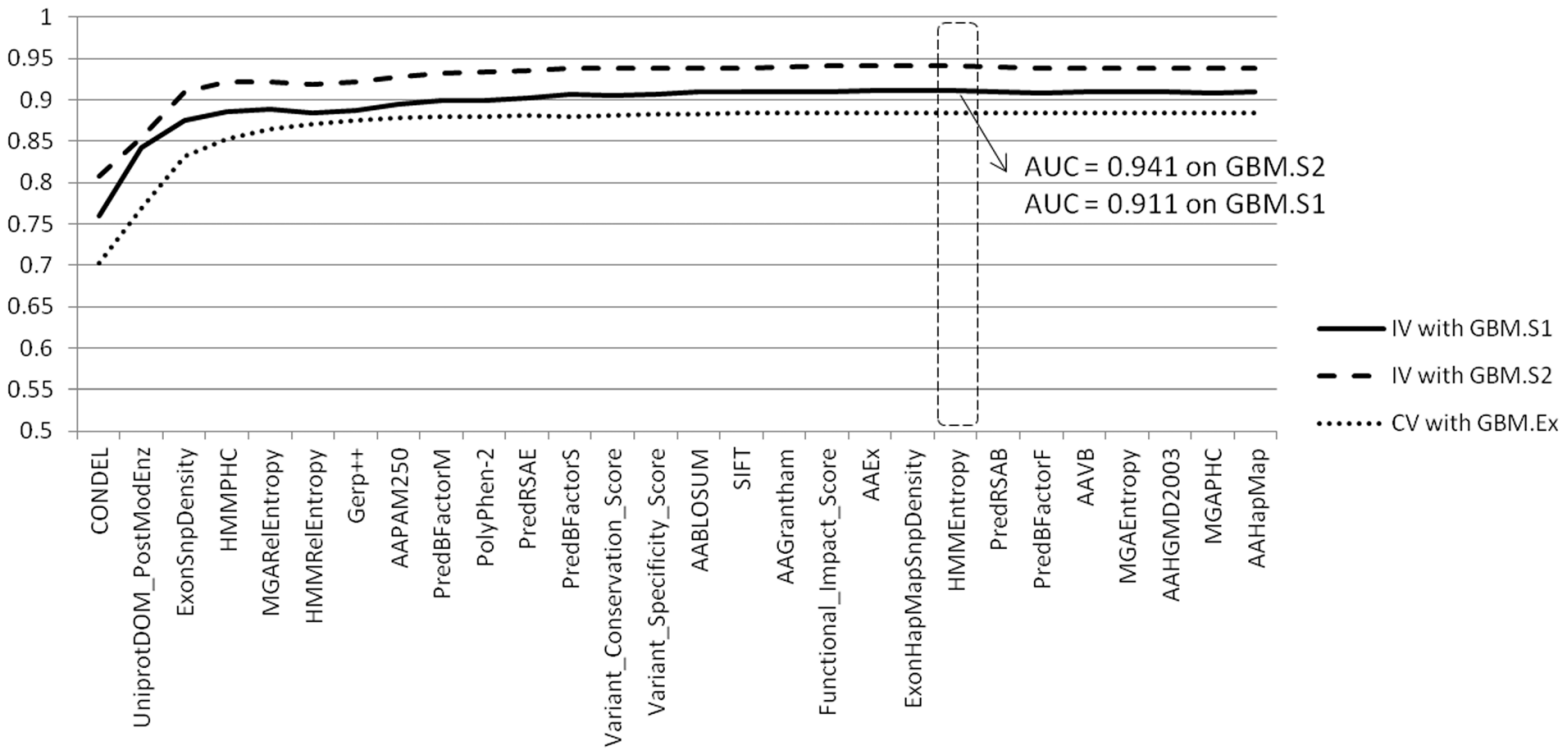
Feature optimization for GBM. Plotted are the areas under the curves (AUCs) of the receiver operator characteristics acquired through our incremental feature selection process. Three sets of AUCs are computed from the 10-fold cross-validation (CV) of the training set GBM.Ex (dotted line) and the independent validation (IV) of 2 test sets, GBM.S1 and GBM.S2 (solid and dashed line). On the x-axis are features that are incrementally selected. The dashed box marks the peaks of the cross-validation AUC, which corresponds to the optimal feature set used for CanDrA.

For GBM, we identified 28 features that individually passed the AUC (|AUC-0.5|>0.08) and Mann-Whitney U test (*P*<0.05 with Bonferroni correction) in the training dataset. These cut-offs were selected to limit the computational burden in the following feature selection. We further identified 3 core features (CONDEL, UniprotDOM_PostModEnz, ExonSnpDensity) and an optimal set of 21 features through our combinatorial feature selection procedure (Materials and Methods, Figure 1, Table S6 in File S1). Among the 3 core features, CONDEL [25], a method that combines five features from SIFT, PolyPhen-2, MutationAssessor and other sources based on a set of 20,000 non-synonymous germline single nucleotide variants (SNVs) was shown to be the single best predictor on the GBM.Ex dataset, with an AUC equal to 0.703. UniprotDOM_PostModEnz (computed by SNVBOX) indicates whether a mutation is located in any enzymatic domain responsible for protein post-translational modification. ExonSnpDensity indicates whether a mutation occurs in a variant-prone exon. Inclusion of these two features further improved the AUC to 0.832 on the GBM.Ex set. This result demonstrated that although general-purpose deleterious SNV prediction tools are applicable to driver prediction, their accuracy could be further improved by including features that are descriptive of the mutational background.

We trained CanDrA using the optimal set of 21 features, and evaluated the performance on the two independent validation datasets (GBM.S1 and GBM.S2). CanDrA achieved AUCs of 0.911 and 0.941, respectively, which compared favorably with those obtained from either CHASM (0.890 and 0.923, respectively) or MutationTastor (0.892 and 0.909, respectively; Table 2).

**Table 2:** Comparisons among 3 tools: CHASM, MutationTastor, and CanDrA.

	CHASM		MutationTastor		CanDrA	
Datasets	AUC	Recall	AUC	Recall	AUC	Recall
GBM.S1	0.890	49/67	0.892	56/67	0.911	56/67
GBM.S2	0.923	44/67	0.909	48/67	0.941	48/67
OVC.S1	0.936	50/61	0.910	46/61	0.953	50/61
OVC.S2	0.940	50/61	0.910	40/61	0.953	47/61

Two metrics are compared: 1) The area under the receiver operator characteristics curve (AUC) for classifying drivers and passengers and 2) the number of drivers predicted by each of the tools vs. the total number of drivers (recall) in each of the 4 test datasets.

**Figure 2:**
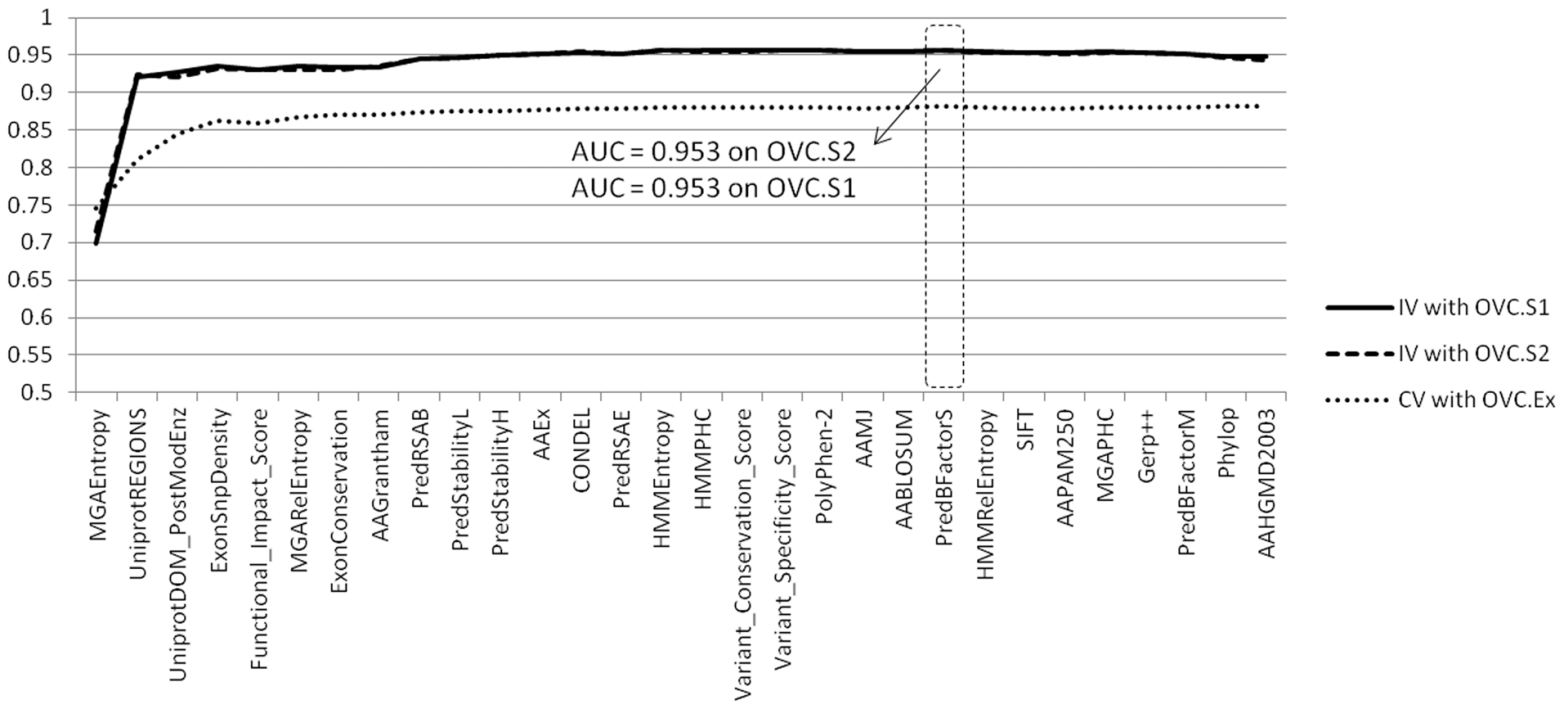
Feature optimization for OVC. Plotted are the areas under the curves (AUCs) of the receiver operator characteristics acquired through our incremental feature selection process. Three sets of AUCs are computed from the 10-fold cross-validation (CV) of the training set OVC.Ex (dotted line) and the independent validation (IV) of 2 test sets, OVC.S1 and OVC.S2 (solid and dashed line). On the x-axis are features that are incrementally selected. The dashed box marks the peaks of the cross-validation AUC, which corresponds to the optimal feature set used for CanDrA.

For OVC, we identified 30 features that individually passed the AUC (|AUC-0.5|>0.05) and Mann-Whitney U test (*P*<0.05 with Bonferroni correction) in the training set. We further identified 3 core features (MGAEntropy, UniprotREGIONS, UniprotDOM_PostModEnz) and an optimal set of 22 features through our combinatorial feature selection procedure (Materials and Methods, Figure 2, Table S7 in File S1). Among the 3 core features, MGAEntropy was the strongest predictor on the OVC.Ex set with an AUC equal to 0.745. It indicates whether a mutation is located in an evolutionarily conserved genomic region and calculates the Shannon entropy from the alignment of homologous proteins in 46 different species [37], [38]. UniprotREGIONS describes functional regions related to protein-protein interaction, biological process regulation, etc. UniprotDOM_PostModEnz for OVC was also selected in the GBM case. These 3 features in combination increased AUCs by >0.06 on the training dataset and >0.2 on the validation datasets.

We trained CanDrA using the 22 features and evaluated its performance on the two independent validation datasets (OVC.S1 and OVC.S2). On both sets, CanDrA achieved AUCs of 0.953, which again compared favorably to those of either CHASM (0.936 and 0.940) or MutationTastor (0.910 on both test sets; Table 2).

### Correlation between CanDrA Scores and Mutation Prevalence

**Figure 3:**
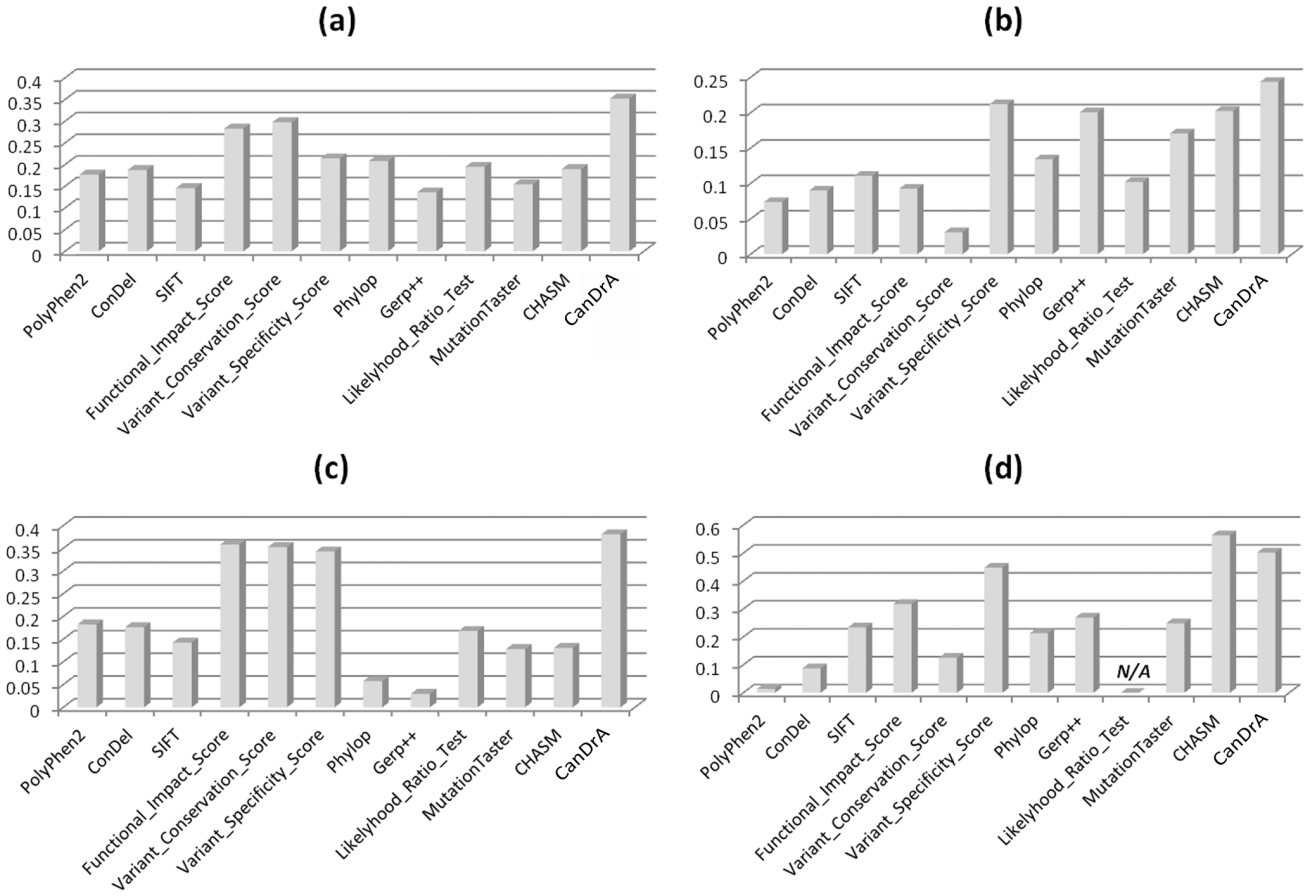
Correlation between mutation score and prevalence. Twelve algorithms (x-axis) were compared using 4 data sets: (a) GBM mutations in *TP53*, (b) GBM mutations in *PTEN*, (c) OVC mutations in *TP53*, and (d) OVC mutations in *KRAS*.

Mutation prevalence, i.e., the frequency of a mutation in a specific cancer type, is a robust indicator of driver functionalities [5], [13], [14], [39]–[42]. If CanDrA is more accurate than other methods, its scores should demonstrate stronger correlation with the mutation prevalence. To test this hypothesis, we created 4 datasets from several most frequently mutated cancer genes: *TP53* and *PTEN* in GBM, and *TP53* and *KRAS* in OVC using data from TCGA and COSMIC (Tables S8–S9 in File S1). We compared the Pearson correlation coefficients between the observed mutation prevalence and the mutation scores of 12 algorithms, in each of the 4 datasets. CanDrA performed better in 47/48 of the comparisons, performing worse only for the one with CHASM using the *KRAS* mutations in OVC (Figure 3). This result clearly indicates the improvement that CanDrA can achieve over the existing methods.

### Predicting Rare Driver Mutations

Of great interest is CanDrA’s capability in predicting drivers that have very low prevalence (e.g., occur only once in a non-hotspot region). The discovery of so-called rare (or tail) driver mutations is a major challenge in current cancer genomics studies but would be of great utility both theoretically and in patient management. Performing a precise assessment of this question requires functional validation data that are currently unavailable for most genes. As a workaround, we used the ratio of driver mutations in known cancer genes as our metric of interest, grounded by the understanding that cancer genes are more likely than non-cancer genes to carry driver mutations [13], [14]. Specifically, we tested whether rare driver mutations predicted by CanDrA are enriched in the COSMIC census cancer genes. We identified rare driver mutations from the COSMIC GBM and OVC mutations that are reported only once, and which have no other mutations in a vicinity of 3 base pairs and were not used as drivers in our training and test sets (Table S10 in File S1). In the 8 known GBM-related genes (*ATM, EGFR, MDM2, MDM4, NF1, PDGFRA, PIK3CA* and *ROS1*), there were 36 GBM mutations, 14 (38%) of which were predicted as drivers by CanDrA. This percentage was significantly higher than the average (13.9%) of the entire set of 1384 mutated genes (p = 3.39×10^−5^, hyper-geometric test). It was also higher than those predicted by other algorithms, except for the variant specificity score of MutationAssessor, which predicted 15/36 (41.7%) drivers (Table S11 in File S1). Among 15 known OVC-related genes (*AKT1, AKT2, ARID1A, BRCA1, BRCA2, CCNE1, CDK12, ERBB2, MLH1, MSH2, MSH6, PIK3R1, PMS2, PPP2R1A* and *STK11*), there were 39 OVC mutations, 22 (56.4%) of which were predicted as drivers by CanDrA. This ratio was significantly higher than the average (20.8%) of the entire set of 5889 mutated genes (p = 2.27×10^−7^, hyper-geometric test). It was also higher than those predicted by other algorithms, including the 19/39 (48%) predicted by CHASM (Table S11 in File S1).

### Discriminating Drivers for Different Cancer Types

A mutation may play different roles in different cancer types (e.g., *BRAF* V600 in colon cancer and melanoma). We examined whether CanDrA can correctly indicate such cancer-type specificity. By combining the 67 and 61 driver mutations from the respective GBM.S1 and the OVC.S1 datasets, we obtained a total of 115 mutations, 41 of which were unique in GBM and 40 in OVC (Table S12 in File S1). For each of the 115 mutations, we computed two scores using CanDrA’s GBM and OVC models, respectively. We observed that mutations found in a specific cancer type scored significantly higher using cancer-type matched models than non-matched models (*p* = 0.0013 for GBM and *p* = 0.0021 for OVC, by Mann-Whitney U test). In addition, mutations unique to a cancer type achieved significantly higher scores using the matched models (*p* = 0.0029 for the mutations unique to GBM and *p* = 0.0138 for the mutations unique to OVC, by Mann-Whitney U test). In all cases, CanDrA achieved more significant discrimination than CHASM (Table 3). Many mutations were associated with different functions in these two cancer types (Table S12 in File S1). For example, the *KRAS* G12V mutation was predicted as a driver in OVC, but as a no-call in GBM. And the *NCOA1* R562G mutation was predicted as a driver in OVC, but as a passenger in GBM.

**Table 3:** Comparison of cancer type specificity between CHASM and CanDrA.

	P value by Mann-Whitney U Test	
Mutation score comparisons between	CHASM	CanDrA
75 GBM and 40 non-GBM mutations using GBM models	0.6093	0.0021
74 OVC and 41 non-OVC mutations using OVC models	0.0350	0.0013
GBM model and OVC model on 41 GBM-specific mutations	0.9114	0.0138
GBM model and OVC model on 40 OVC-specific mutations	0.8323	0.0029

Each row represents a comparison of 2 groups of scores computed from the mutations and the models listed in column 1.

### Comparison Using Real Data versus Synthetic Data

**Figure 4:**
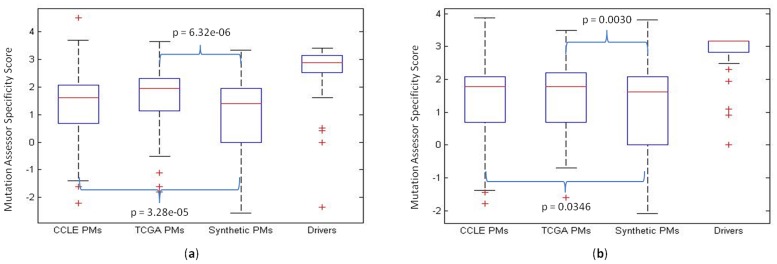
Comparison between synthetic passenger mutations (PMs) and real PMs. Plotted are the Mutation Assessor variant specificity scores of sets of synthetic PMs (generated by CHASM), CCLE PMs, TCGA PMs and driver mutations from the 4 test sets in Table 1, for GBM (a) and OVC (b), respectively. Significant differences (Mann-Whitney U test) between two score distributions are indicated with *P* values reported.

We suspected that CanDrA’s better performance over that of CHASM could be partially attributed to its use of real passenger mutations (RPMs) instead of SPMs in training the models. We believed that although SPMs can reflect certain mutagenic characteristics of a cancer type (e.g., exposure to environmental mutagens), they are likely insufficient in representing other factors such as evolutionary conservation, sequence context, and protein domains. To gain deeper insight, we performed two experiments. First, we compared the RPMs with the SPMs in terms of their variant specificity scores (VSC), functional impact scores (FIS) and variant conservation scores (VCS) computed by MutationAssessor. These scores, especially VSC, were among the most predictive features in our stringent validation (Figures S2–S3 in File S1). The distributions of these scores indicated that the RPMs were significantly more deleterious than SPMs for both GBM and OVC, and therefore are likely better examples for distinguishing real drivers from passengers. Using VSC, the differences among the distributions of RPMs, SPMs and drivers were shown in Figure 4. Similarly significant results were observed using VCS and FIS. Second, we trained CHASM to classify identical number of RPMs and SPMs from the same set of drivers. CHASM performed considerably worse with RPMs (AUC = 0.907 for GBM and 0.938 for OVC, on average) than with SPMs (AUC = 0.943 for GBM and 0.949 for OVC).

## Discussion

Our investigation resulted in a new software tool, CanDrA, which was demonstrably more accurate than other tools in predicting cancer-type-specific driver mutations. We have pre-computed CanDrA scores for almost all possible (around 77 million) missense mutations across whole genome in several major cancer types and enabled users to perform very efficient predictions using desktop computers or servers. Due to the vast amount of missense mutations and the low throughput of existing functional experiments, even small improvements in prediction accuracy can lead to dramatically better efficiency and cost savings in validating driver mutations.

One important distinction between CanDrA and other methods is the inclusion of a very large set (95) of features, collected from almost all available methods. Although this ensures the comprehensiveness of CanDrA, it also increases the difficulty of deriving an optimal model due to the “curse of dimensionality” (COD), i.e., it requires exponentially more samples to train a robust model with increased number of features [43]. The SVM method used by CanDrA is more robust against the COD than other classifiers, including the random forest algorithm used by CHASM [44]. Moreover, the two-step feature selection approach that we applied effectively alleviated COD while maintaining the interpretability of the results, which makes it more advantageous than other exhaustive, filter-based, or transformational methods [45].

Our feature selection results shed some light on the similarity and dissimilarity between GBM and OVC that may be driven by different mutagenic mechanisms. For example, high grade serous ovarian cancer has almost universal mutation of *TP53* and approximately 50% have aberrations predicted to alter DNA repair through homologous recombination, as compared to GBM that has much higher frequency of aberrations in pathways related to cell signalling. For both cancer types, we found that a mutation is more likely to be a driver if it occurs on residues that are evolutionary conserved, have stiff backbones, or have less solvent accessibility; although more drivers occur in evolutionarily conserved residues in OVC than in GBM (Figures S2–S3 in File S1). On the other hand, features that represent protein domain knowledge, such as UniprotDOM_PostModEnz and UniprotREGIONS, seem to convey more specific information on cancer type. In our stringent sets, a considerable portion (50%) of GBM drivers are located in protein enzymatic domains responsible for post-translational modification (indicated by UniprotDOM_PostModEnz), contrasted by around 7% of GBM passengers, 5% of OVC drivers, and 6% of OVC passengers. Around 70% of OVC drivers are located in protein domains that may mediate protein-protein interactions or other biological processes (indicated by UniprotREGIONS), contrasted by around 5% of OVC passengers, 24% of GBM drivers, and 6.3% of GBM passengers. These results underscore the importance of performing cancer-type-specific feature selection and modeling in achieving accuracy.

Constructing a representative training dataset is of ultimate importance to approaches that depend on supervised training. For this reason, we performed an exhaustive curation of cancer mutation data from COSMIC, TCGA, and the CCLE project and defined putative drivers and passengers based on their observed frequency. The low prevalence of driver mutations and the relatively small number of samples in a single cancer type currently available has made it difficult to derive sufficient numbers of examples for supervised training. We alleviated this problem by utilizing data from other cancer types, as motivated by CHASM and the knowledge that many driver mutations occur in more than one type of cancer [46]–[49]. It is possible that such expanded data curation processes may reduce cancer type specificity, which is difficult to quantify without having sufficient functional validation data. A similar argument may apply to our selection of passenger mutations, which was based on mutation prevalence and existing knowledge of cancer genes. For some cancer types, such as melanoma, the mutation rate is so high that it may be inaccurate to define drivers and passengers based only on prevalence. Despite these potential pitfalls, the consistency between the cross-validation and the independent validation results and the superior performance of CanDrA over other methods suggest that our strategy of constructing the training dataset is beneficial overall. Clearly, as more functionally validated driver mutation data become available from large-scale functional assays such as those in the Cancer Target Discovery and Development (CTD^2^) Network, we will be able to refine our training set and further improve the accuracy of CanDrA.

## Supporting Information

File S1 **Supplementary Tables and Figures. Figure S1.** Definition of CanDrA score, category and significance. **Figure S2.** Evaluation of single descriptors by ROC AUC on GBM.S1 and GBM.S2. Shown are descriptors with |AUC-0.5|>0.1 and P value (Bonferroni corrected) <0.05. **Figure S3.** Evaluation of single descriptors by ROC AUC on OVC.S1 and OVC.S2. Shown are descriptors with |AUC-0.5|>0.1 and P value (Bonferroni corrected) <0.05. **Table S1.** Missense mutations in set GBM.S1. **Table S2.** Missense mutations in set GBM.S2. **Table S3.** Missense mutations in set OVC.S1. **Table S4.** Missense mutations in set OVC.S2. **Table S5**. Ninety-five (95) features involved in CanDrA modeling. **Table S6.** Feature combination selected for GBM in CanDrA. **Table S7.** Feature combination selected for OVC in CanDrA. **Table S8.** GBM missense mutations for testing correlation between CanDrA scores and mutation prevalence. **Table S9.** OVC missense mutations for testing correlation between CanDrA scores and mutation prevalence. **Table S10.** Rare missense mutations from GBM and OVC related genes. **Table S11.** Comparison of algorithms for predicting rare driver mutations. **Table S12.** Missense mutations used for evaluating CanDrA and CHASM in predicting cancer-type-specific drivers.
(XLS)
